# Solid-contact ion-selective electrode for the determination of silver ions released from silver sulfadiazine in sodium hyaluronate formulations: evaluation of whiteness and greenness profiles

**DOI:** 10.1186/s13065-025-01546-7

**Published:** 2025-06-19

**Authors:** Manal Ibrahim, Nesrin K. Ramadan, Magda M. Ibrahim, Shereen A. Boltia

**Affiliations:** 1 Egyptian Drug Authority, Giza, Egypt; 2 Analytical Chemistry Department, Faculty of Pharmacy, Cairo University, Cairo, Egypt

**Keywords:** Silver sulfadiazine, Potentiometric determination, Multi-walled carbon nanotubes screen-printed electrode, Greenness and whiteness evaluation

## Abstract

Potentiometric sensors were designed with a focus on rapid, environmentally friendly, cost-efficient, and highly specific detection. These sensors were specifically tailored for the analysis of silver ions released from silver sulfadiazine (SSD) in combination with sodium hyaluronate (SH) in their combined dosage form. The manufacturing process involved a two-step optimization procedure. Initially, various ionophores were evaluated to enhance the selectivity of the sensors, with Calix [4]arene demonstrating the highest affinity for silver ions. The inclusion of a cation-exchanger in the membrane ensured selective response toward cations namely, Ag⁺ ions from SSD thereby exhibiting permselectivity. In the second optimization phase, a layer of multi-walled carbon nanotubes (MWCNTs) was incorporated between the Calix[4]-containing polymeric membrane and the solid-contact screen-printed electrode (SPE). This MWCNT layer served as an ion-to-electron transducer, improving potential stability by mitigating drift. This stability enhancement is likely due to its hydrophobic nature, which prevents the formation of a water layer at the interface between the electrode surface and the polymeric sensing membrane. The sensor, developed in accordance with IUPAC recommendations, exhibited high selectivity for Ag⁺ ions from SSD in the presence of SH in the pharmaceutical dosage form. The MWCNT-modified sensor achieved high accuracy (99.94% ± 0.413), a linear response in the concentration range of 1.0 × 10⁻⁵ to 1.0 × 10⁻² M, and a detection limit of 4.1 × 10^− 6^ M. The slope, calculated from the linear portion of the calibration curve, was found to be 61.029 mV/decade, indicating near-Nernstian behavior. To evaluate the environmental and health implications of the proposed method in comparison to a previously reported technique, comprehensive assessment tools including the Analytical Eco-scale, GAPI, AGREE, and RGB12 model were employed for greenness and whiteness profiling.

## Introduction

The analytical technique of ion-selective electrode (ISE) relies primarily on recording the electric potential generated from movement of different ions in the aqueous layers [1]. In contemporary analytical practices, there is a preference for direct methods to swiftly determine pharmaceuticals while adhering to the principles of green analytical chemistry. The ISE method stands out as a straightforward and environmentally friendly analytical approach for directly determining drugs, eliminating the necessity for sample extraction or purification. Its application extends across various fields, including biological, environmental, and medical settings [1].

The first developed ion-selective electrodes (ISEs) were the liquid-contact ISEs, employing internal solutions with the sensing membrane positioned between the inner filling solution and the sample solution [2]. However, these liquid-contact electrodes come with inherent restrictions, like the risk of liquid leakage and challenges in miniaturization. In contrast, solid-contact ion-selective electrodes (SC-ISEs) are preferred over their liquid counterparts, featuring simplicity, stable and reproducible responses, ease of handling, and long-term storage capabilities. Additionally, SC-ISEs are more appropriate to the bulk fabrication of cost-effective sensors, making them particularly advantageous for the related techniques [3, 4]. A prominent type of SC-ISE is the screen-printed electrode (SPE) [5]– [6], which replaces traditional all solid-state ISEs like the classical coated-wire electrode [7] and glassy carbon or metallic electrodes [8–10].

However, SPEs have exhibited some drawbacks, including unplanned modifications in the arrangement of screen-printed elements when encounter certain aqueous solutions [11]. Water layer was probably formed internally and factors like CO_2_, light, pH and oxygen may affect the sensitivity of the SPE [12]. To tackle these challenges, solutions have been sought by incorporating single-walled carbon nanotubes [11, 13–15] and multi-wall carbon nanotube powder [16] into SPEs. This incorporation aims to enhance signal strength and electrode stability while eliminating undesired water layers.

**Fig. 1:**
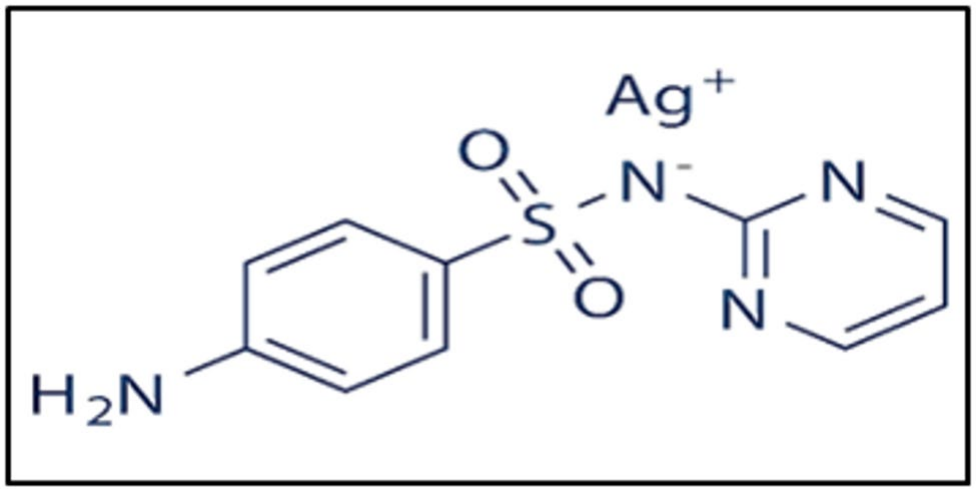
Structure of silver sulfadiazine

Silver sulfadiazine (SSD) (Fig. 1) is a sulfa antibiotic used to prevent, manage, and treat burn wound infections. The combination of SSD with SH in one dosage form promotes the wound healing process, as sodium hyaluronate binds to many water molecules, thereby ensuring the hydration of the skin and connective tissue [17].

Numerous analytical methods have been reported for the determination of SSD in its pure form and pharmaceutical formulations, including high-performance liquid chromatography [18–24], spectrophotometric techniques [25–30], and more recently, electrochemical methods [31–33]. While HPLC and spectrophotometric methods offer high sensitivity and selectivity, they often require expensive instrumentation, complex sample preparation, use of hazardous organic solvents, and long analysis times. These limitations can hinder routine or on-site analysis, especially in resource-limited or environmentally sensitive settings.

In contrast, electrochemical sensors, particularly ion-selective electrodes, offer significant advantages such as rapid response, simplicity, low cost, miniaturization potential, and environmental friendliness. Among these, screen-printed electrodes (SPEs) have gained increasing attention due to their disposable nature, low production cost, and suitability for mass fabrication. However, to the best of our knowledge, screen-printed solid-contact ISEs have not been previously employed for the potentiometric determination of silver ions released from SSD in pharmaceutical formulations.

This study aims to fill this gap by developing and optimizing a microfabricated solid-contact ISE based on SPEs, incorporating selective ionophores and a multi-walled carbon nanotube (MWCNT) transducer layer for the green, accurate, and reliable detection of SSD in topical formulations.

Monitoring silver sulfadiazine (SSD) is essential due to its widespread use as a topical antimicrobial agent in burn and wound treatments. Ensuring the accurate dosage of SSD in pharmaceutical formulations is critical for maintaining therapeutic efficacy while minimizing potential side effects such as silver toxicity or bacterial resistance. Reliable quantification also supports quality control during manufacturing, ensures regulatory compliance, and enables stability studies for product shelf-life evaluation. Therefore, the development of a simple, selective, and environmentally friendly method for SSD analysis, such as the proposed potentiometric sensor, is highly relevant and necessary for both clinical and industrial applications.

In this study, a two-step optimization strategy was employed to develop solid-contact ion-selective electrodes (ISEs) with high selectivity and stable potential response for the target analyte silver ions released from silver sulfadiazine (SSD). The first phase involved the fabrication of six liquid-contact electrodes, each incorporating a different ionophore, to evaluate their selectivity toward SSD. This screening process aimed to identify the ionophore that provided the most favorable potentiometric performance. In the second phase, two solid-contact electrodes were constructed using screen-printed electrodes (SPEs) coated with a polyvinyl chloride (PVC)-based sensing membrane containing the selected ionophores. Among the six molecular recognition ionophores tested, the membrane incorporating calix[4]arene (CX4) exhibited the most desirable performance in terms of sensitivity, selectivity, and signal stability, making it the optimal choice for further sensor development.

CX4 was utilized to create two SPE_s_, one coated solely with the CX4 membrane (sensor 7), and the other initially coated with a layer of multi-wall carbon nanotube before applying the CX4-doped PVC sensing membrane (MWCNT SP sensor 8). MWCNT serves as an efficient ions-to-electron transducer, forming a hydrophobic layer upon contact with specific ions. This property prevents the significant formation of an aqueous film at the interface between the underlying electrode and the sensing membrane, enhancing sensor performance and increasing electrical signal stability.

The stability and performance of the designed sensor were assessed through water layer test and signal drift applications, to study the effect of MWCNT inclusion. The MWCNT sensor demonstrated high stability, selectivity, sensitivity, and high accurate assessment of SSD without the need for extraction from its pharmaceutical formulation.

There is currently a significant emphasis among analysts on adopting green analytical chemistry (GAC). The primary goal of greenness strategies is to utilize smaller quantities of chemicals, minimize exposure to vapors and emissions, all while maintaining the accuracy of analytical methods [34]. An effective tool for assessing greenness is the Eco-Scale assessment procedure [35], which gives penalty points for the utilized reagent types and amounts according to their hazard degree to environment and human, energy consumption by electrical devices, and the approach to analytical waste treatment.

Whiteness of analytical procedures, a concept developed in 2021 to assess the maintainability of analytical methods, employs the red-green-blue 12 algorithm (RGB-12) based on the principles of white analytical chemistry (WAC) [36]. Various greenness assessment tools, including the Green Analytical Procedure Index (GAPI) [37] and Analytical Greenness Metric (AGREE) [38], were applied to evaluate the environmental impact of analytical procedures. These tools contribute to a comprehensive understanding of the ecological footprint associated with analytical methods, allowing for informed and environmentally conscious choices in the field of analytical chemistry.

## Experimental

### Instruments

A pH/mV meter, specifically Jenway (Model 3505) from the UK, was employed in this study. The system utilized an Ag/AgCl double-junction type external reference electrode, specifically the Thermo Scientific Orion 900,200 from MA, USA. The internal filling solution of the electrode was comprised of 1.0 × 10^− 3^ M of KCl solution saturated with AgCl, while the external filling solution comprised 10% KNO3. Additionally, a Jenway pH glass electrode (Type 3505, UK) was utilized for pH adjustments. To facilitate mixing, a magnetic stirrer manufactured by Sonorex from Hungary was incorporated into the experimental setup. This combination of instruments and electrodes provided a comprehensive platform for the precise analysis and adjustment of ion concentrations in the study.

### Materials

#### Standard materials

A standard material of silver sulfadiazine (SSD) was kindly supplied by EDA (Egyptian Drug Authority), Egypt, and its purity was found to be 99.71% ± 0.65 when checked against a reported method [25].

#### Pharmaceutical formulations

Medihyalo cream (batch number 212687), labelled to contain 10 mg/g silver sulfadiazine and 2 mg/g sodium hyaluronate, manufactured by (DBK for pharmaceutical industries), Cairo, Egypt.

### Chemicals and reagents

#### Chemicals

All chemicals were of analytical grade and the water was double distilled.

Polyvinylchloride (PVC) of high molecular weight, Tetrahydrofuran (THF), 2- Nitrophenyl octyl ether (NPOE), Sodium tetrakis [3,5-bis (trifluoromethyl) phenyl] borate (Na. Tetrakis), 4-tert-Butylcalix[8]arene, Calix[6]arene, Calix[4]arene, C-Undecylcalix[4] resorcinarene monohydrate, Cucurbit[6]uril hydrate, 2-hydroxypropyl-β-cyclodextrin (β-CD). Glacial acetic acid and potassium chloride (KCl), Sigma-Aldrich (Germany). Boric acid; Fischer chemicals (UK), o-phosphoric acid; El Nasr Pharmaceutical Co (Egypt), sodium hydroxide; LOBA Chemie (India), carbon screen-printed electrodes (SPE) 3 mm diameter was provided from CH instruments; Inc.; (TX, USA) These electrodes were used as received, without any further surface modification or fabrication, multi wall carbon nano-tube (MWCNT); Fluka (Steinheim, Germany), with (Outer diameter of 10–20 nm, length of 1–10 μm, purity of around 95% and number of walls of 3–15 walls).

#### Reagents

- (1.0 × 10^− 3^ M) of KCl solution.
- The Britton–Robinson (BR) buffer was prepared by mixing equal volumes of 0.04 M glacial acetic acid, 0.04 M phosphoric acid, and 0.04 M boric acid, resulting in a final concentration of 0.013 M for each acid component. The pH was adjusted in the range of 3–10 using 0.2 M NaOH and o-phosphoric acid [39].
- Dilute ammonia solution (3.3%): 10.0 mL ammonia solution (33%) from Fisher chemical (UK) and complete to 100 mL with water.

### Solutions of standards

- Stock standard solution of SSD (1.0 × 10^− 2^ M) was prepared by dissolving 178.6 mg SSD with 25 mL diluted ammonia solution (3.3%) in a 50-mL volumetric flask, sonicated for 30 min for complete dissolving. Britton Robinson buffer (pH 5) was used to complete the volume.
- Working standard solutions of SSD: Series of sequential dilutions were transferred from the stock standard solution into a set of 10-mL volumetric flaks, and the volume was completed with Britton-Robinson buffer pH 5 (1.0 × 10^− 5^ to 1.0 × 10^− 3^ M) to be used as calibration solutions.

### Procedures

#### Fabrication of membranes

To facilitate a comparative analysis, six distinct PVC-membrane sensors were created, employing 2-nitrophenyl octyl ether (NPOE) as a plasticizer. The chosen cation exchanger was sodium tetrakis [3,5-bis (trifluoromethyl) phenyl] borate (Na.Tetrakis), and it was paired with six different ionophores, namely, 4-tert-Butylcalix[8]arene (Calix[8]), Calix[6], calix[4], C-Undecylcalix[4] resorcinarene monohydrate (Calix[4] resorcinarene), Cucurbit[6]uril hydrate (Cucurbit[6]uril), and 2-hydroxypropyl-β-cyclodextrin (β-CD).

The membrane preparation process involved combining 190 mg PVC and 10 mg Na.Tetrakis in six glass petri dishes with 5-cm diameter. Subsequently, 10 mg of each ionophore was individually added to one petri dish. The contents of each petri dish were mixed with 0.4 mL NPOE and dissolved in 4.0 mL THF to create homogeneous membranes. To achieve a thickness of 0.1 mm, filter paper was used to cover the petri dish, allowing excess THF to evaporate. The petri dishes were then left undisturbed overnight to yield PVC master membranes. The relative weight percentages (w/w%) of the membrane components were PVC ∼ 30.1%, Na-TFPB ∼ 1.6%, ionophore ∼ 1.6%, and NPOE ∼ 66.7%.

#### Liquid contact (LC) electrodes assembly

By using a cork borer to cut a rounded disk approximately 9 mm diameter from every one of the six recently constructed PVC master membranes, then each disk was attached to the end of flexible PVC cylinder which was fixed to a suitable hard plastic tube with THF to produce six sensors. The inner-filling reference solution was constructed by adding 1.0 × 10^− 3^ M of SSD to 1.0 × 10^− 3^ M of KCl in equal volumes, then filling the PVC cylinder part in each sensor with this solution. The internal reference electrode was finished by immersing a wire of Ag/AgCl about 1-mm in diameter in the inner-filling reference solution.

#### Preparation of MWCNT solution

A stock solution of multi-walled carbon nanotubes (MWCNTs) was formulated by using 4 mL of tetrahydrofuran (THF) to dissolve 15 mg of MWCNTs and 0.5 mL of o-NPOE by the aid of sonication for 20 min to achieve a consistent suspension. Then 2.5 mL was transferred from the stock solution to a 25-mL volumetric flask, then THF was used to complete the volume to the mark to produce the working solution of MWCNTs. To ensure uniform dispersion, the working solution underwent sonication for 5 min each time before usage.

#### Solid contact (SC) electrodes assembly

**Fig. 2:**
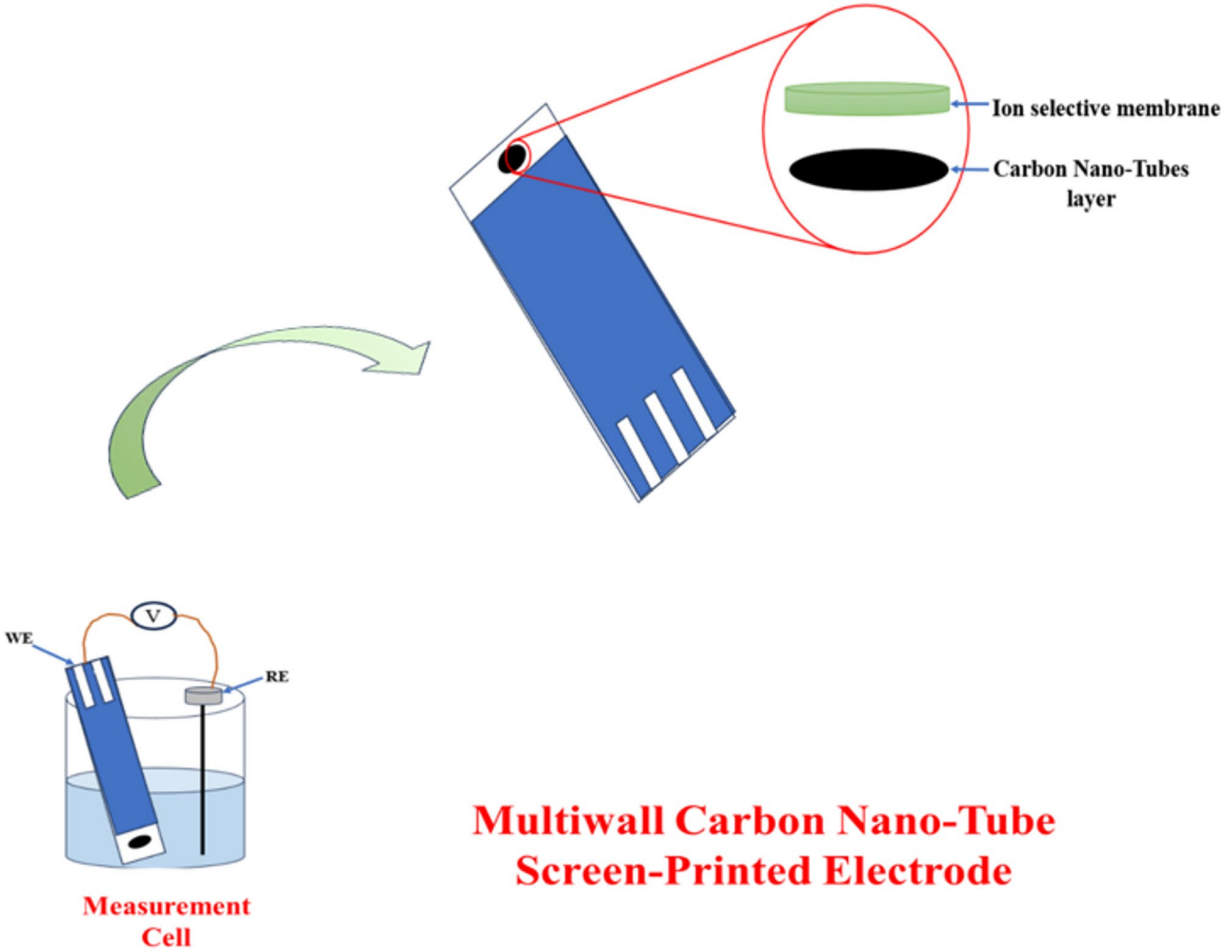
Multiwalled carbon nanotube screen printed sensor (MWCNT-SP) and screen printed (SP) sensor

At first, 6 mL of tetrahydrofuran (THF) was used to mix and dissolve the constituents of the ion-selective membrane (ISM) containing CX4 as ionophore. Afterward, a precise 10 µL of the resulting uniform blend was delicately applied by drop-casting onto two screen printed electrode sensor 7 & 8. To improve electrical signal stability and mitigate potential drift in the ion-selective membrane (ISM), the screen-printed electrode (sensor 8) underwent an initial coating with 10 µL of the MWCNT working solution. The coated electrode was left overnight to enable solvent evaporation before the introduction of the ISM blend. The two fabricated electrodes (sensors 7 & 8) Fig. 2 were allowed to dry at room temperature for one day.

#### Effect of pH on electrode response

Solutions of SSD concentrations of 1.0 × 10^− 3^ and 1.0 × 10^− 4^ M were prepared in two sets with Britton-Robinson buffer. In each set, the pH values range was adjusted to (3–10) by 0.1 M NaOH. The obtained potential from each solution was recorded by using the fabricated 7 & 8 sensors.

#### Calibration of the electrodes

SSD drug solutions in concentrations (1.0 × 10^− 6^ to 1.0 × 10^− 2^ M) were first prepared with Britton-Robinson buffer (pH 5.0) and then transferred to 10-mL beakers. Each electrode was immersed in the SSD solutions alongside a double junction of Ag/AgCl reference electrode. A magnetic stirrer was used to keep equilibrium in the solutions helping to produce stable performance of the potentiometer. The electromotive forces (emf) were registered to within ± 1 mV for liquid contact (LC) sensors (1–6), ± 0.5 mV for SPE (sensor 7) and 0.1 mV for MWCNT SPE (sensor 8). The electrodes were rinsed with Britton-Robinson buffer (pH 5.0) between measurements. By relating the obtained emf from the developed sensors and–log molar concentrations of SSD, the calibration graphs were constructed. While the Nernst equation defines the response in terms of ion activity, the use of molar concentration in calibration graphs is a standard practical approximation under dilute conditions, where the activity coefficient is close to unity. The regression equations were computed from the linear part of the graphs.

#### Assessment of stability by water layer test and signal drift monitoring

This test involved assessing the behavior of the screen-printed electrodes (sensors 7 & 8) over a three-hour period. Potentiometric readings were documented for each sensor during the initial hour in a 1.0 × 10^− 3^ M SSD solution. Subsequently, in the second hour, an interfering 1.0 × 10^− 2^ M sodium hyaluronate solution was introduced, and the electromotive force (emf) was obtained. The test concluded by returning to a 1.0 × 10^− 3^ M SSD solution for the third hour. Continuous monitoring of the signal drift was checked by observing the emf behavior every 2 min during the first hour.

#### Determination of SSD in pharmaceutical formulation

The amount of Medihyalo^®^ cream, claimed to contain 17.86 mg of SSD, was weighed and transferred into a 50-mL volumetric flask. Subsequently, 30 mL of dilute ammonia solution was used to dissolve, and complete dissolution was obtained after sonication for 30 min. Britton-Robinson buffer (pH 5) was used to complete the volume to create a solution of 1.0 × 10^− 3^ M SSD. By using sensors 7 & 8, alongside with the double-junction Ag/AgCl reference electrode, the produced potential from the prepared solution was recorded. Regression equation for each sensor was applied to calculate the SSD concentration.

For further analysis, 1.0 × 10^− 4^ M solution of SSD was prepared by diluting 1 mL of 1.0 × 10^− 3^ M of SSD solution with Britton-Robinson buffer to 10 mL and its corresponding potential was measured. By applying the corresponding regression equation, the SSD concentration was obtained.

#### Effect of interfering substances on the electrode selectivity

The selectivity of sensors 7 & 8 towards different substances found in the dosage form with SSD was determined by applying the separate solutions method [40]. The produced potentials of the sensor were recorded, and the selectivity coefficients were calculated.

## Results and discussion

The fabrication of stable and reproducible solid-contact ion-selective electrodes (SC-ISEs) typically requires a multi-step development process [41]. In this study, the primary objective was to construct a miniaturized SC-ISE that offers high sensitivity, reproducibility, and potential stability for the selective determination of silver ions released from SSD. To achieve this, a two-step optimization strategy was implemented. In the first step, liquid-contact electrodes incorporating various ionophores were evaluated to identify the most suitable ionophore for SSD recognition. In the second step, the selected ion-selective membrane was applied onto a screen-printed (SP) solid-contact electrode. To further minimize potential drift and enhance signal stability, a layer of multi-walled carbon nanotubes (MWCNTs) was introduced between the SP electrode and the sensing membrane. This hydrophobic transducer layer prevents the formation of an undesirable aqueous layer at the interface, thereby improving electrode performance.

### Screening and selection of ionophores

The composition of the developed ISEs affects directly on its performance. Using a PVC matrix in the fabrication process plays a pivotal role in trapping the ion association complexes in the ISEs. A crucial component influencing ISE performance is the plasticizer employed. Solvent mediators’ “Plasticizers” regulate membrane permeability by adjusting the distribution of the ion-exchangers, thereby optimizing selectivity and sensitivity. It is essential to optimize the proportion of the plasticizer used to decrease the electrical asymmetry of the membrane and prevent leaching of its components into the aqueous phase [42]. In this study, NPOE was chosen as the plasticizer for all sensors because of its high dielectric constant. This property helps to increase the mobility of ions and permeability of the membrane, facilitating proper diffusion of SSD throughout the membrane.

The selection of ion exchangers is very critical in the fabrication of ISEs. These hydrophobic ionic sites facilitate ion-exchange kinetics at the membrane-substrate interface, effectively reducing resistance in bulk by introducing mobile ionic sites into the membrane matrix [43]. To enhance the performance characteristics of a membrane sensor, the inclusion of an ionophore into the PVC matrix proves beneficial. This addition improves selectivity and sensitivity by forming host-guest complexes with the intended analyte, consequently decreasing the analyte’s detection limit, and enhancing the determination selectivity. Calix[n]arenes are widely employed as supramolecular hosts in the composition of ion-selective membranes. These molecules feature electron-rich basket-shaped cavities that exhibit a preference for accommodating large drug molecules. Acting as selective ligands, calixarenes form dipole-dipole interactions thus establishing cation interactions with various ions. The binding strength between the membrane and the target is influenced by the size of the calixarene cavity [44].

Cyclodextrins, on the other hand, present cage-shaped structures with hydrophobic centers capable of encapsulating diverse drug molecules [45]. In their application, the primary intermolecular interactions involve hydrogen bonds, hydrophobic interactions, and Van der Waals forces, contributing to cooperative binding processes. Cucurbiturils find utility as an efficient host molecule in supramolecular recognition within the field. Their notably high affinity for positively charged or cationic compounds is thought to be facilitated by hydrophobic interactions and cation-dipole interactions [46].

Six lipophilic ionophores HP-β-cyclodextrin (HP-β-CD), calix[4]arene (CX[4]), calix[6]arene (CX[6]), calix[8]arene (CX[8]), calix[4] resorcinarene, and cucurbit[6]uril were investigated in combination with the cation exchanger sodium tetrakis[3,5-bis(trifluoromethyl)phenyl]borate (Na-TFPB) to fabricate six distinct ion-selective electrodes (ISEs). Each sensor was individually calibrated, and the slope and intercept were derived from the linear portion of the calibration curve. The detection limit (LOD) was calculated according to the IUPAC definition [47].

All fabricated sensors demonstrated near-Nernstian responses, indicating effective complexation with silver ions released from silver sulfadiazine (SSD) Table 1. Among them, Sensor 1 based on CX[4] exhibited a slope of 60.37 mV/decade, while HP-β-CD showed a comparable slope of 60.29 mV/decade. Although the numerical differences were modest, CX[4] was selected as the optimal ionophore due to its slightly lower detection limit, higher intercept, and greater reproducibility across replicates.

To support this selection, a one-way analysis of variance (ANOVA), followed by Tukey’s post hoc test, was performed on the slope data. While the difference between CX[4] and HP-β-CD was not statistically significant (*p* > 0.05), CX[4] outperformed the other macrocyclic ionophores with statistically significant margins (*p* < 0.05). The superior performance of CX[4] is attributed to its rigid, preorganized cavity structure, which likely facilitates stronger and more selective complexation with Ag⁺ ions. In contrast, the more flexible and open structure of β-CD may result in weaker and less consistent binding, accounting for the slightly reduced sensitivity and reproducibility observed.

### Validation of sensors

Validation of the selected sensor and its electrochemical characteristics were comprehensively done according to IUPAC requirements [47], as detailed in Table 2.

**Table 1:** Effect of different combinations of ionophores with a cation exchanger in the fabricated PVC sensors on the slope, intercept and detection limit of SSD

Sensor	Ion exchanger	Ionophore	Slope (mV/decade)	Intercept (mV)	Detection limit (mol/L)	Number of replicates	Average ± RSD
**1**	Na.Tetrakis	Calix[4]	60.37	420.53	3.1 × 10^− 5^	6	99.96 ± 0.834
**2**	Na.Tetrakis	Calix[6]	59.12	415.59	2.5 × 10^− 5^	6	99.92 ± 1.51
**3**	Na.Tetrakis	Calix[8]	56.17	410.13	2.2 × 10^− 5^	6	99.92 ± 1.15
**4**	Na.Tetrakis	Calix[4] resorcinarene	54.97	417.23	4.5 × 10^− 5^	6	99.95 ± 0.673
**5**	Na.Tetrakis	Cucurbit[6]uril	55.32	408.49	4.7 × 10^− 5^	6	99.93 ± 1.35
**6**	Na.Tetrakis	β-CD	60.29	418.71	3.6 × 10^− 5^	6	99.93 ± 0.966

**Table 2:** Electrochemical response characteristics and validation parameters of the proposed sensors 7 & 8 for the determination of SSD

Parameter	Sensor 7	Sensor 8
**Slope (mV/decade)**	59.257	61.029
**S.E. of slope**	0.0199	0.0094
**Intercept (mV)**	536.44	368.27
**S.E. of intercept**	0.0453	0.01
**Correlation coefficient (r)**	0.9997	0.9999
**Concentration range (M)**	1.0 × 10^− 5^– 1.0 × 10^− 2^	1.0 × 10^− 5^– 1.0 × 10^− 2^
**LOD (M)**	3.0 × 10^− 6^	4.1 × 10^− 6^
**Response time (s)**	15	11
**Working pH range**	(5 ± 0.5)	
**Stability (weeks)**	8	8
**Accuracy** ^a^ **(% Mean ± SD)**	100.29 ± 0.315	99.94 ± 0.413
**Repeatability** ^b^ **(RSD%)**	0.265	0.490
**Intermediate precision** ^c^ **(RSD%)**	0.769	0.933

^[a]^ Average of determination of three concentrations (*n* = 9)

^[b]^ Repeatability: the intraday precision (*n* = 9), average of three concentrations repeated three times within the day

^[c]^ Intermediate precision: the intraday precision (*n* = 9), average of three concentrations repeated three times on three consecutive days

### Screen printed electrodes using CX4-Na tetrakis membrane

In response to the growing demand for disposable solid-contact ion-selective electrodes (SC-ISEs), which are increasingly favored over traditional liquid-contact designs, the CX[4] membrane was ultimately selected as the ionophore for the fabrication of two solid-contact sensors dedicated to the determination of silver ions released from silver sulfadiazine. The first sensor (Sensor 7) consisted of a screen-printed (SP) electrode modified solely with the CX[4]-based membrane. The second sensor (Sensor 8) incorporated an additional thin interlayer of multi-walled carbon nanotubes (MWCNTs) between the SP electrode and the CX[4] membrane, serving as an ion-to-electron transducer to enhance potential stability Fig. 2.

**Fig. 3:**
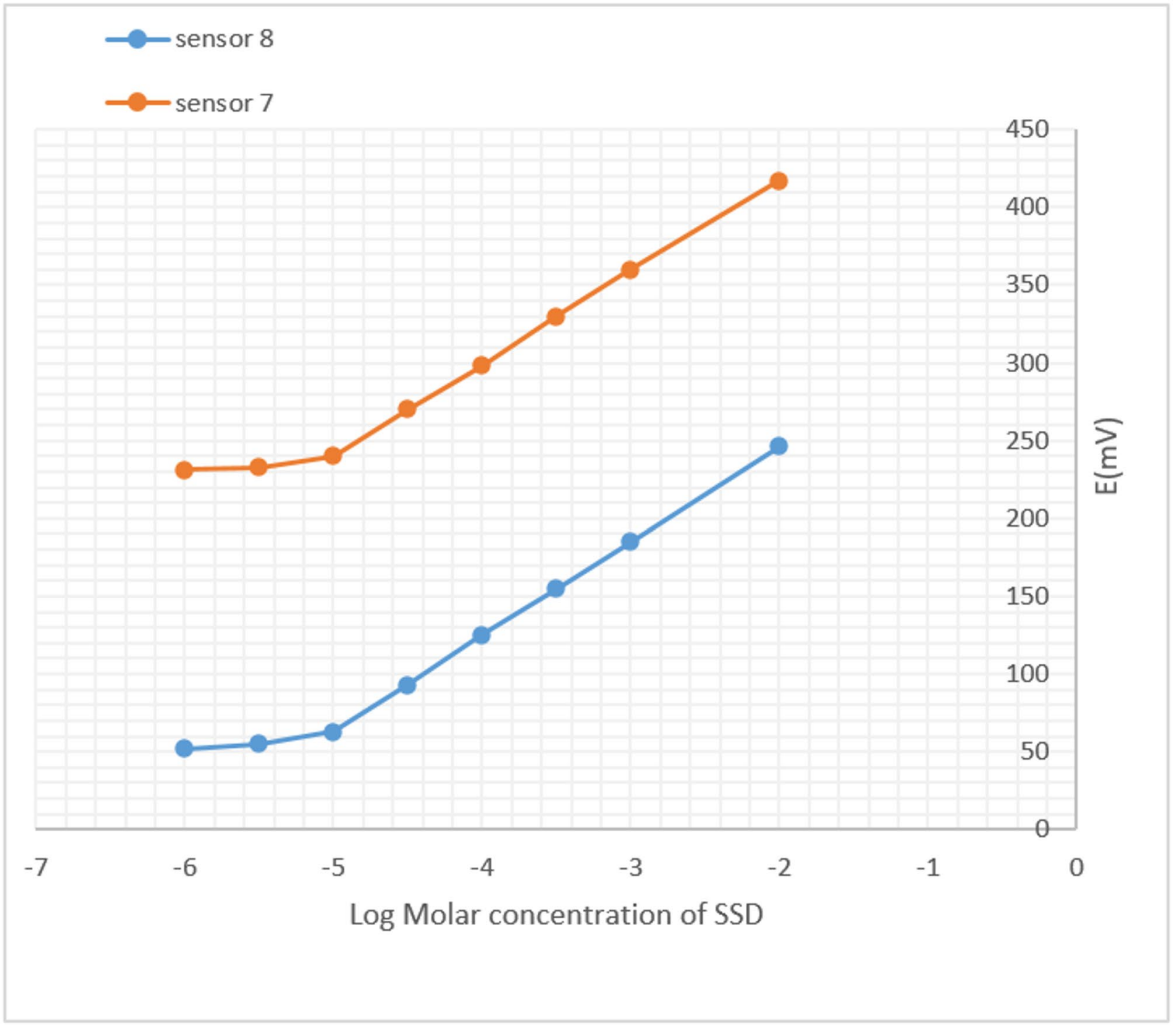
Calibration profiles showing the potential (mV) versus log [SSD], reflecting the response to Ag⁺ ions released from SSD using Sensor 7 and Sensor 8

Both Screen Printed sensors (Sensors 7 and 8) exhibited Nernstian responses over the concentration range of 1.0 × 10⁻⁵ to 1.0 × 10⁻² M, with slopes of 59.257 mV/decade for Sensor 7 and 61.029 mV/decade for Sensor 8, as presented in Table 2. The corresponding calibration curves are shown in Fig. 3.

### Effect of pH

**Fig. 4:**
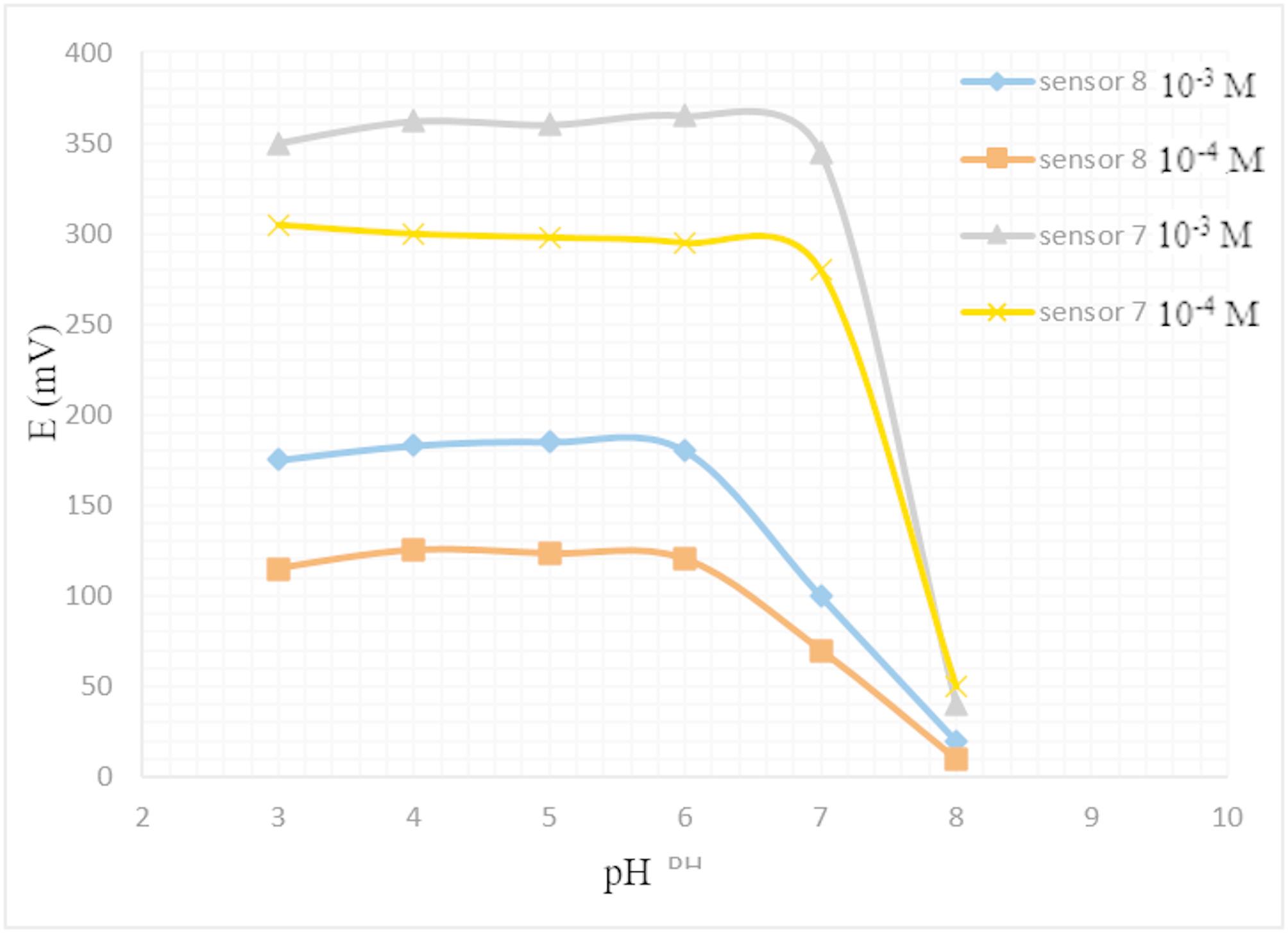
Effect of pH on the response of Sensor 7 & Sensor 8

The effect of pH on the performance of the most stable MWCNT-modified screen-printed sensor (Sensor 8) was examined, as shown in Fig. 4, to determine the optimal pH conditions for accurate detection of Ag⁺ released from silver sulfadiazine. The sensor exhibited a stable and consistent potential response in the pH range of 4.0 to 6.0, where the release of Ag⁺ ions from SSD is favored, and the drug remains soluble and ionized. The pKa of sulfadiazine (6.36) supports the use of phosphate buffer at pH 5.0 as the optimal medium, providing a balance between solubility and ion availability.

Above pH 6.0, a gradual decrease in potential was observed, likely due to reduced release of free Ag⁺ ions and potential complexation or precipitation phenomena. At pH ≥ 8.0, the precipitation of silver compounds and/or SSD was observed, resulting in a marked decline in sensor response due to reduced Ag⁺ ion availability. At pH values below 4.0, increased signal noise and instability were detected, possibly due to interference from excess protons and changes in membrane behavior under highly acidic conditions.

### Water layer test and signal drift monitoring for the proposed SPE_s_

The formation of an aqueous layer at the interface between the solid-contact and the ion-selective membrane is known to cause potential drift and reduce long-term stability of solid-contact ion-selective electrodes. Although the MWCNT-modified sensor exhibited favorable short-term potentiometric behavior, the possibility of water layer formation beneath the membrane could compromise its long-term stability.

To evaluate this effect, a potentiometric water layer test was performed following the standard protocol [48]. Initially, the potential was measured in a 1.0 × 10⁻³ M solution of silver sulfadiazine, from which Ag⁺ are released into solution. The sensor was then immersed in 1.0 × 10⁻² M sodium hyaluronate solution, which contains no free silver ions, to test for any significant potential change. A temporary decrease in potential was observed. However, upon re-immersion in the original SSD solution, the potential rapidly returned to its initial value. This recovery suggests that no persistent water layer had formed beneath the membrane, as such a layer would have altered the internal ionic environment, resulting in noticeable and irreversible potential drift.

**Fig. 5:**
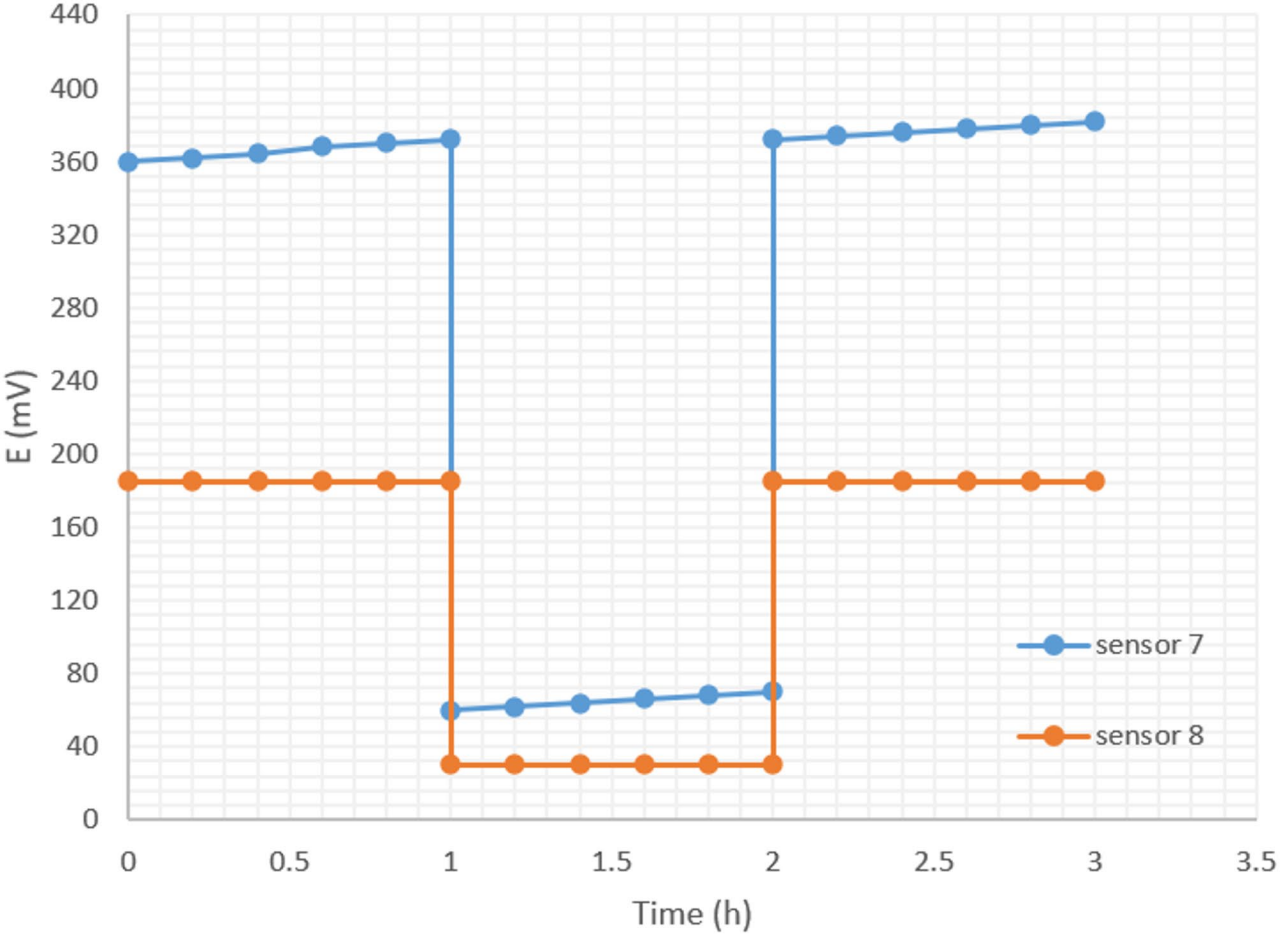
Water layer test for Sensor 7 & Sensor 8. Measurements were recorded in 1.0 × 10^− 3^ M SSD and 1.0 × 10^− 2^ M sodium hyaluronate

As illustrated in Fig. 5, the incorporation of MWCNTs as an ion-to-electron transducer significantly enhanced the sensor’s stability. The MWCNT-containing sensor (Sensor 8) showed minimal drift of + 1 mV/h, while the unmodified sensor (Sensor 7) exhibited a more pronounced drift of + 10 mV/h, confirming the beneficial role of MWCNTs in maintaining a stable solid-contact interface.

### The selectivity of the proposed sensors

The specificity of SSD-ISEs was assessed in comparison to other compounds present in the medication formulation through the distinct solutions technique [40]. This involved creating 1.0 × 10^− 3^ M solutions of sodium hyaluronate, propylene glycol, glycerol, sodium methyl, and propyl parabens. The computed selectivity coefficients indicated that the suggested sensors exhibit a high level of specificity for SSD, without noteworthy interference from other substances, as detailed in Table 3.

**Table 3:** Potentiometric selectivity coefficients of the proposed sensors

Interferent (1 × 10^− 3^ M)	Sensor 7	Sensor 8
**Sodium hyaluronate**	5.6 × 10^− 3^	1.7 × 10^− 3^
**Propylene glycol**	1.9 × 10^− 3^	4.4 × 10^− 3^
**Glycerol**	3.2 × 10^− 3^	3.9 × 10^− 4^
**Propyl paraben sodium**	3.4 × 10^− 3^	5.1 × 10^− 3^
**Methyl paraben sodium**	3.3 × 10^− 3^	5.2 × 10^− 3^

### Potentiometric determination of SSD in pharmaceutical preparation

The proposed sensors were successfully applied for the determination of silver ions released from SSD in pharmaceutical preparation Medihyalo^®^ cream, labeled to contain 10 mg/g of SSD. The results presented in Table 4 represent the percentage recovery relative to this labeled content. For example, a recovery of 100.3% indicates that the measured concentration of SSD closely matches the expected value, confirming the accuracy of the method.

**Table 4:** Determination of SSD in Medihyalo ^®^ cream and statistical comparison of the results of dosage form with the reported method

Parameters	Pharmaceutical preparation		
	Sensor 7	Sensor 8	Reported method [25] ^a^
**Mean ± SD**	100.3 ± 0.63	100.1 ± 0.49	99.72 ± 0.61
**n**	6	6	6
**% RSD**	0.628	0.488	0.607
**Variance**	0.397	0.240	0.372
**student’s t-test (2.228)** ^**b**^	1.68	1.25	-
**F value (5.05)** ^**b**^	1.067	1.55	-

^[a]^ Reported method was HPLC method using an RP- column C18 (250 mm X 4.0 mm, 5 μm) at 30ºC, and mobile phase consisted of 0.1% phosphoric acid: acetonitrile (90: 10) at flow rate 1 ml/min and λ = 265 nm

^[b]^ These values represent the corresponding tabulated values of *t* and *F* at *p* = 0.05

The findings demonstrated high precision and excellent concordance with those obtained using a previously reported HPLC method [25]. Statistical analysis using the t-test and F-test at a 95% confidence level (*p* = 0.05) revealed no significant differences in terms of accuracy or precision between the proposed potentiometric sensors and the reference chromatographic method, further validating the reliability and applicability of the developed sensors for pharmaceutical analysis.

While six replicate measurements were performed on a single batch of the cream to ensure precision, we acknowledge that further validation using multiple batches and different product lots would enhance the assessment of the method’s robustness and applicability in routine quality control settings.

### Greenness assessment for the proposed sensor

Several green assessment methods have been applied to conform if the analytical procedures applied adhere to the requirements of green analytical chemistry (GAC), aimed at promoting sustainability and reducing environmental impact [49]. One such method is the Eco-scale, which involves assigning penalty points to assess the method’s alignment with green chemistry principles. Another method is the RGB12 algorithm, which assesses the overall sustainability of the analytical process through four different colors. Additional evaluation tools like AGREE and GAPI have been utilized to offer full analysis of the analytical procedures according to GAC principles. Employing multiple green tools is recommended to encompass all aspects of GAC principles, as each tool emphasizes different aspects, providing deeper insights for a more thorough assessment of greenness of the applied method [50].

#### Analytical eco-scale method

This approach relies on assigning penalty points based on the type and quantity of reagents utilized during the analysis process and how much waste is produced during the analytical process. The method employs a mathematical equation that subtracts the total penalty points from 100. The evaluation of the analytical method then depends on the outcome of this mathematical process, with a perfect green process having an Eco-Scale value of 100. As the value decreases, it indicates a less environmentally friendly and less desirable analytical procedure [35]. Table 5 illustrates the penalty points for both the developed and the reported methods [25]. The developed method shows a high Eco-Scale score, while the reported method exhibits a poor green score attributed to the extensive usage of harmful organic solvents.

**Table 5:** Penalty points for the greenness assessment of the proposed potentiometric method as compared with those of the reported method

Parameter	Penalty points	
	Proposed method	Reported method [24]
**Reagents**		
Methanol	0	3
Ethanol	0	3
Acetonitrile	0	3
Phosphoric acid	8	8
Ammonia	3	0
**Instruments**		
Energy	1	6
Occupational hazard	0	8
**Waste**	4	16
**Total penalty points**	16	47
**Score**	84	53

#### Assessment of whiteness by the (RGB12 algorithm) tool

This is a sustainable development tool that encompasses the environment, society, and economy within the analytical procedure. It is used to evaluate the analytical methods quantitatively by computing the “whiteness” of the method [51]. In this model, the red color is associated with analytical efficiency, incorporating validation parameters like accuracy, precision, LOD, and sensitivity. The green component represents adherence to GAC requirements about environmental safety, including reagent toxicity, quantity and type of reagents used, waste generation, energy consumption, and overall environmental impact. The blue component concerns the economic part and the production yield, like time, expenses consumed, practical necessity and how the method is simple.

The RGB12 algorithm depends on completing the three colors in an excel template spreadsheet, resulting in an automatic calculation and assessment. The resulting color depends on the proportion of each primary color. The resulting white color is characterized to the ideal analytical method with high sustainability. The excel spreadsheet provides a simultaneous assessment, where a score of 0 indicates the unfavorable result, and 100 indicates that the method is more desirable for the analytical method when regarding specific principles [52].

**Fig. 6:**
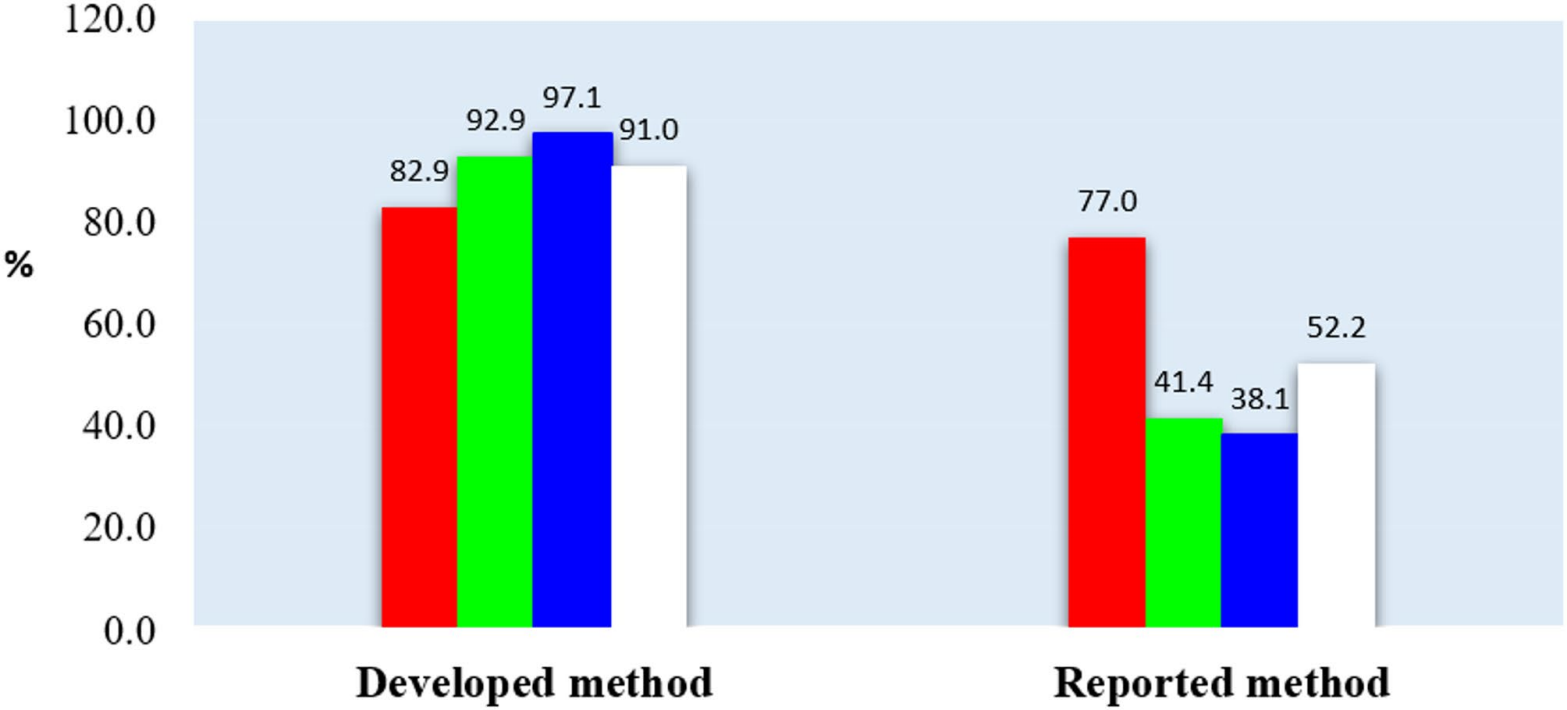
The RGB 12 algorithm comparing between the developed and the reported RP-HPLC method used for determination of SSD

Upon comparing the reported PR-HPLC method [25] with the proposed MWCNT SP-ISE method using the RGB12 metric, Fig. 6 reveals significant discrepancies in the results across the three colors. The proposed ISE method achieved a substantial 97.1% score in the blue color category, surpassing the reported HPLC method’s score of 38.1%. This percentage underscores the considerable economic impact of the ISE method, attributed to its reduced consumption of reagents, energy, and time utilized during the analytical procedure.

In terms of the red color, the proposed ISE method obtained a higher score, reflecting its superior validity across all analytical parameters of the analysis procedure. It demonstrated greater precision, sensitivity, and accuracy compared to the reported method.

The green color assessment showed the proposed method, scoring 92.9%, while the reported method scored 41.4%. This notable difference in scores was linked to the reduced usage of reagents and solvents in the proposed method, contrasting with the substantial amounts of acetonitrile and methanol utilized in the reported method’s analytical procedure. Consequently, the proposed method emerged as the less toxic and hazardous option for both the environment and human health.

The whiteness score for the proposed method stood at an impressive 91%, whereas the reported method scored 52.2%. An overall examination of the results across the four colors generated by the RGB12 algorithm for both methods indicated consistently higher percentages for the proposed method. This confirms its heightened greenness, superior whiteness results, and enhanced overall validity compared to the reported method. The comprehensive evaluation underscores the economic and environmental advantages of the proposed MWCNT SP-ISE method.

#### Green analytical procedure index tool (GAPI)

This tool systematically assesses the environmental impact of each stage in the analytical procedure, adhering to (GAC) requirements. It evaluates various aspects such as transportation, sampling, preparation of samples, storage, and the utilization of solvents and reagents throughout the analytical process. The resulting output is a pictogram consisting of a pentagon with four additional pentagons positioned on the edges, excluding the base. Different colors, including green, yellow, and red, are assigned to represent low, medium, and high environmental impacts, respectively, for each step in the analytical process.

**Fig. 7:**
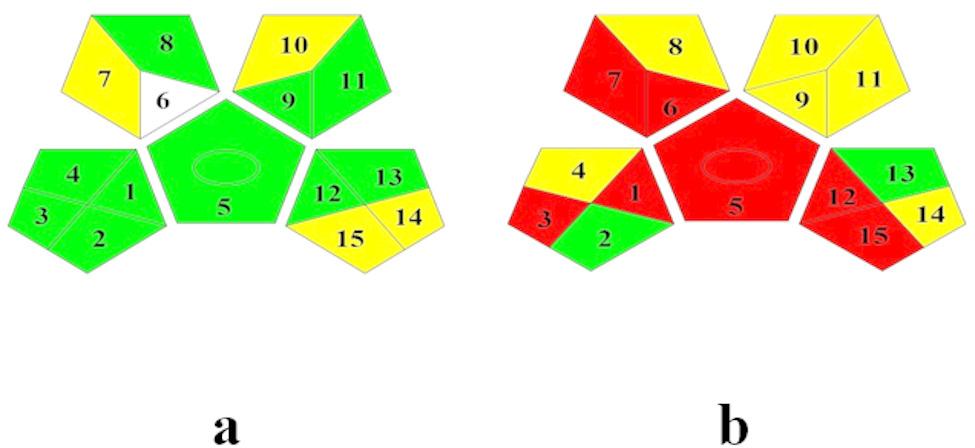
Green Analytical Process Index (GAPI) pictograms of the developed ISE method **(a)** and the reported HPLC method **(b)**

Figure 7 displays pictograms with distinct results. For parameter 1, related to data collection, the developed method is represented in green, as it allows for in-line data collection, contrasting with the reported method that employs off-line data collection. Parameters (3, 4) focusing on transport and storage show green for the developed method, indicating minimal use of chemicals or reagents with specific precautions. In contrast, the reported method shows red and yellow, signaling the use of acetonitrile, methanol, and phosphoric acid, requiring special precautions during transport and storage.

Parameters (5, 6, 8) highlight the simplicity of the developed method, while the reported method involves numerous preparation steps, including extraction and treatment, resulting in lower scores depicted in red and yellow. Parameters (7, 9, 12) assess the amounts of reagents and solvents used, with the reported method showing higher consumption (10 mL per sample), increased energy use, and greater waste generation per sample.

Parameters (10, 11) pertaining to reagents and solvents reveal that the reported method requires large amounts of acetonitrile and methanol, contributing to higher scores for health and safety hazards, represented in yellow. The comprehensive visual representation provided by the GAPI tool facilitates the comparison of procedures between different methods, aiding in the selection of the more environmentally preferable approach.

#### Greenness analytical tool (AGREE)

The AGREE (Analytical greenness) tool, considered the most widely used greenness evaluation method, employs a circular symbol with twelve sections, each representing one of the twelve concepts of Green Analytical Chemistry (GAC). The color of each section signifies the achievement of the corresponding GAC concept, with dark green indicating success (1 score) and red indicating non-compliance (0 score). The colors combination and average scores for the twelve sections is presented as both a color and number in the center of the AGREE symbol [53].

**Fig. 8:**
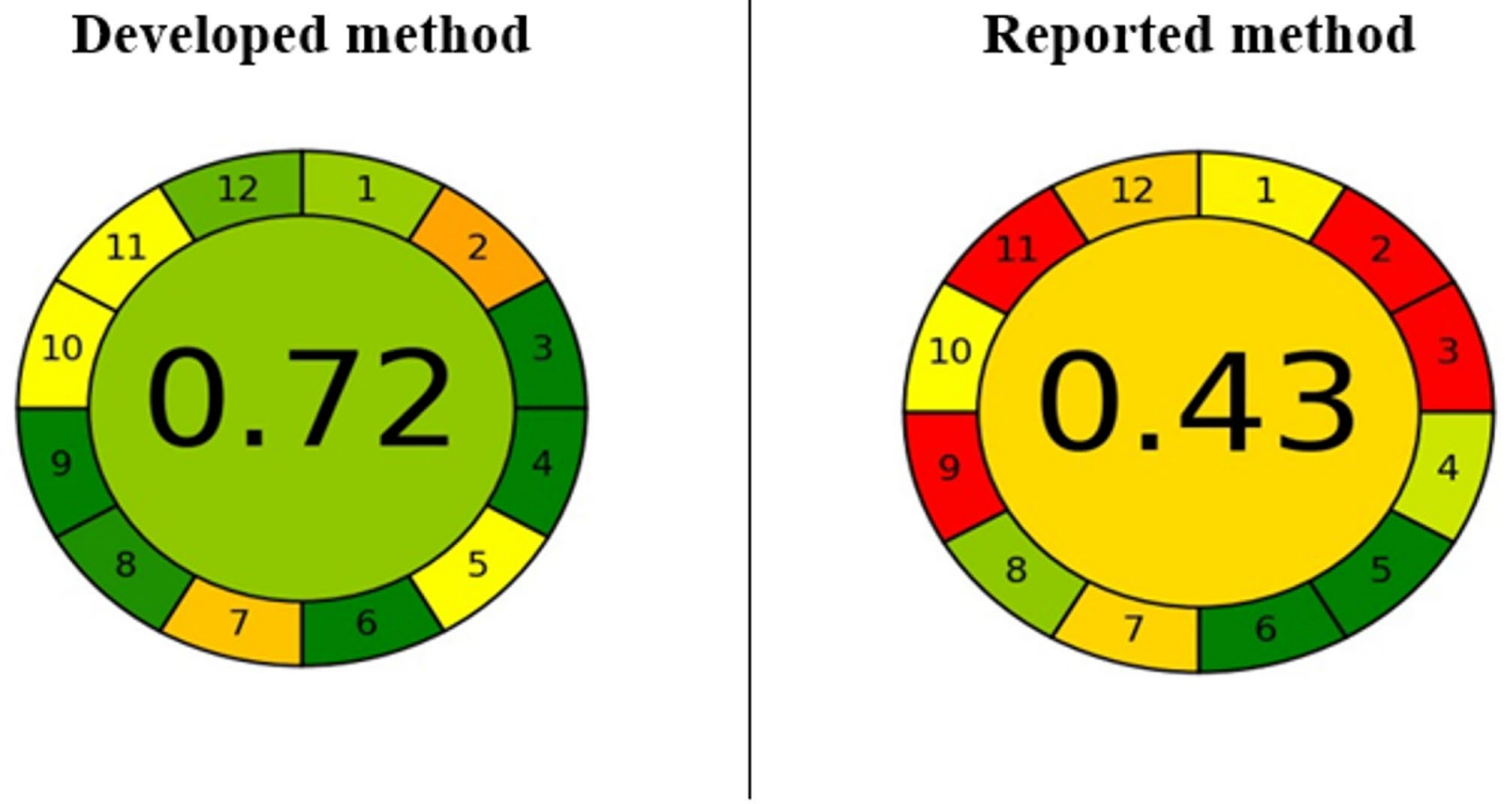
The AGREE symbols results for the developed and reported methods

In Fig. 8, the AGREE symbols for both the proposed and reported methods are depicted. Sections 1 and 3 show green for the developed method due to in-line sampling, while yellow and red are seen for the reported method due to off-line sampling. Section 2 displays orange for the proposed method and red for the reported method, reflecting the reduced reagent requirement in the developed method. Section 4, representing the number of preparative steps, shows dark green for the developed method and apple green for the reported method, as the developed method involves three steps or fewer.

Section 5 reveals dark green for the reported method because of the automatic sampling procedure and downsized, while the developed method shows yellow due to manual electrode use.

Based on the AGREE symbol results in Figure. 8, the developed method, with a score of 0.72, seemed to be more greener coming from lower utilization of solvents and energy and less generation of waste. In contrast, the reported method scores 0.43, indicating less greenness resulting from higher solvent and energy consumption, along with increased waste production.

In conclusion, the comparison between the developed and the reported methods in assessment of greenness demonstrates the superiority of the developed method. The developed method stands out for its lower consumption of chemical reagents, reduced energy consumption, absence of vapor emissions in the environment, and minimal health and safety hazards. These findings are consistent with the better results observed in the Eco-scale, RGB12, and GAPI evaluations, affirming that the developed method is more preferred than the reported method from the point of maintainability and environmental impact.

## Conclusion

The presented results demonstrate the successful development of a promising microfabricated solid-contact ion-selective electrode (SC-ISE) specifically tailored for the determination of silver ions released from silver sulfadiazine. The use of calix [4]arene as the ionophore significantly enhanced both the sensitivity and selectivity of the sensor. Further improvements were achieved by employing a solid-contact configuration, which was optimized through the incorporation of a multi-walled carbon nanotube layer serving as an ion-to-electron transducer. The MWCNT interlayer effectively mitigated the common issue of potential drift in SC-ISEs, which typically compromises signal reproducibility. Its hydrophobic nature minimized the formation of an unwanted water layer at the interface between the transducer and the polymeric sensing membrane, thereby enhancing the sensor’s long-term stability and overall performance compared to conventional ion-selective electrodes. The developed microfabricated sensors align with current advancements in portable and miniaturized analytical devices, offering advantages such as ease of mass production, cost efficiency, and suitability for routine applications. They provide a practical, robust alternative to traditional methods, eliminating the need for extensive sample pretreatment, hazardous organic solvents, or expensive analytical kits. In addition to performance validation, this study introduced an environmentally conscious evaluation through the assessment of greenness and whiteness using tools such as the Analytical Eco-scale, GAPI, AGREE, and RGB12. This holistic approach highlights not only the technical innovation of sensor design but also its contribution to sustainable, eco-friendly analytical practices underscoring the novelty and broader significance of the work within the field of green analytical chemistry.

## Data Availability

The datasets used and/or analysed during the current study are available from the corresponding author on reasonable request.
